# Positive Association Between Therapeutic Alliance and Quality of Life in Psychodynamic Psychotherapy, Cognitive Behavior Therapy, and Interpersonal Therapy: The Patient's Perspective

**DOI:** 10.3389/fpsyt.2021.613627

**Published:** 2022-01-25

**Authors:** Guilherme Kirsten Barbisan, Luiza Zamban de Pieri, Leonardo Gonçalves, Cinthia Danielle Vasconcelos Rebouças, Neusa Sica da Rocha

**Affiliations:** ^1^Postgraduate Program in Psychiatry and Behavioral Sciences, Hospital de Clínicas de Porto Alegre, Universidade Federal do Rio Grande do Sul, Porto Alegre, Brazil; ^2^Universidade Federal de Ciências da Saúde de Porto Alegre, Porto Alegre, Brazil

**Keywords:** psychotherapy, quality of life, therapeutic alliance, therapeutic relationship, outcome

## Abstract

**Background:**

The therapeutic alliance (TA) is considered a common psychotherapeutic factor associated with positive results in psychotherapies. There are no studies relating the TA with quality of life (Qol).

**Objectives:**

Our objective was to evaluate whether there is an association between the TA and Qol across three different psychotherapies.

**Methods:**

A cross-sectional study, which included outpatients undergoing individual psychotherapeutic treatment was conducted. When analyzing the total sample, the correlation of the TA with Qol domains did not present statistical significance. When considering only the sample of patients who were undergoing treatment in psychodynamic psychotherapy (PP), there was a statistically significant association between the TA and the psychological domain of Qol (*p* < 0.05). When using a regression model for adjusting for confounding factors, the association between psychological domain with the TA on the PP patients sample lost significance (*p* = 0.221).

**Discussion:**

These findings suggest that the TA seems to be more strongly related to better QoL in PP.

## Introduction

Psychotherapies are important tools in the treatment of many mental disorders. A systematic review of 61 meta-analyses (1) (852 clinical trials out of a total of 137,126 participants) that examined the effect of pharmacotherapy and psychotherapy for major psychiatric disorders showed that the effect sizes of psychotherapies tended to be higher [0.58; 95% confidence interval (CI) 0.40–0.76] than those of pharmacotherapy (0.40; 0.28–0.52).

Nowadays there are several different models of psychotherapy, however, in this study we will focus on three specific techniques: interpersonal psychotherapy (IPT), cognitive-behavioral therapy (CBT), and psychodynamic psychotherapy (PP), which are the three amongst commonly used types of psychotherapies.

IPT was developed to treat depression in the 1970s (2), and assumes the development of depression and anxiety in an interpersonal context, including its inception and response to treatment and outcomes, which are formed by relationships involving the individual and anyone in that person's sphere. The interpersonal approach examines the connections between relationships with others. The goal of IPT is to expand the patient's social network; the therapist is an important support system in the beginning, but should not assume this role. As such, shorter treatments meet this goal and encourage the patient to strengthen external support networks.

CBT was developed by Beck and Alford (3), in the late 1960s, and is a psychotherapeutic method based on the cognitive model, according to which emotion and behavior are influenced by the way the individual interprets events. Although the central element in understanding the individual's problems is cognition, CBT recognizes the reciprocal interaction between thoughts, emotions, behaviors, physical reactions, and the environment.

PP has broader goals. In addition to the reduction of symptoms, the patient is expected to develop greater awareness of his interpersonal, intrapersonal, and personality difficulties, among others. This scope is due to the way in which the therapy is structured, focusing on the effects and expression of emotions, the exploration of feelings and thoughts, the identification of repetitive patterns, the discussion of past experiences, the understanding of interpersonal relationships and the relationship with the therapist, and the study of desires and fantasies (4).

Psychotherapies are performed in the context of an interpersonal relationship or a therapeutic relationship, which depends on both the patient's and therapist's characteristics in order to establish and sustain such treatment (5). Some of these factors are known as non-specific factors, Roger's factors (6), or common factors and are determinants of the results of all therapies.

Among so-called non-specific factors, the therapeutic alliance (TA) has been the subject of much study, with numerous instruments developed to analyze it. For decades, the TA has been one of the most studied factors that lead to the success of psychotherapy (7), and today, there are more than 30 scales that have been created to measure the TA in the psychotherapeutic context.

The TA has been found to predict the outcome of a variety of different psychotherapies (8), and it has been described as “the quintessential integrative variable” in psychotherapy (9). Recent studies corroborate the evidence that a good TA positively influences the outcome of psychotherapy (10). TA research is welcome as an example of an area of research with direct relevance to psychotherapeutic practice (11). A positive TA has demonstrated increased retention in substance abuse treatment and improved treatment outcomes, regardless of the time of treatment (12, 13).

In a recent meta-analysis (7) conducted to evaluate TA and treatment outcomes, independent studies including more than 30,000 patients (published between 1978 and 2017) were included for face-to-face and Internet-based psychotherapy. The overall association of outcomes and TA for face-to-face psychotherapy was r = 0.278 [95% CI (0.256–0.299); *p* 0.0001, equivalent to d 0.579]. This effect size is for the third decimal place identical to that found in the 2011 meta-analysis (r 0.278) (14). The overall effect size of 0.278 indicates that the alliance–result relationship accounts for about 8% of the variability in treatment results. The correlation for Internet-based psychotherapy was approximately the same (r 0.075, k 23).

These results confirm the robustness of the positive relationship between the TA and treatment outcomes. In this meta-analysis, the vast majority of included trials focused on depressive and anxious symptoms as an outcome. No study used scales that assessed quality of life (QoL) as a measure of outcome that was correlated to the TA. In addition, this study shows that there is a disproportionate collection of data from North America (k 208), English-speaking countries (k 21), and European countries (k 65). No study from Brazil or other South American countries was included. A study conducted by Thomas et al. (15), in the oncology area, with patients with advanced cancer participating in a primary palliative care intervention, shows that trusting relationships between patients with advanced cancer and their oncologists are associated with better patient quality of life. A recent study examined the influence of the TA on QoL in a brief seven-session CBT program for OCD (16) and used the 12-Item Short-Form Health Survey (SF-12) which includes only mental and physical components of the Qol, which is different to the WHOQOL-Bref measure that includes psychological, physical, social and environmental domains. No statistical association was found.

Evidence that associates the TA as a predictor of symptomatic improvement (mainly depressive and anxious symptoms) in psychotherapy treatments is already reasonably broad, but there are no studies associating the TA as a predictor of improvement in patients' QoL as the outcome. This association seems of particular importance since many patients seek psychiatric/psychotherapeutic care, not only to reduce symptoms but also to improve their satisfaction with their personal life in general (work, family, affective).

In addition, there is evidence that psychiatric disorders, such as depression, have a considerable and widespread impact on a patient's life, not just on the symptomatology of the disease. A study conducted by Fleck et al. (17) showed that the intensity of depressive symptoms is inversely related to various subjective indicators of well-being and health. This study showed that patients with depressive symptoms had more physical and psychological impairment and rated their quality of life as worse (measured by two World Health Organization Quality of Life assessment tools [WHOQOL-BREF] generic quality of life issues).

There have been many studies that used sophisticated methodologies and naturalistic designs (18) which used the Patient Estimate of Improvement (19), a 16-item questionnaire assessing improvement during psychotherapy across a broad range of patient functioning beyond only symptomatic change. It is not precisely a measure of quality of life and it cannot be used in other fields, which impairs the comparisons of its results when analyzing different types of treatments including, for example, medication.

In view of this definition, our first hypothesis postulates that there is a positive association between TA and QoL in treatments in the three different modalities of psychotherapy. Also, we hypothesize that this association will be greater in PP since the patient-therapist relationship is specifically analyzed in treatment sessions as part of its therapeutic process. The primary objective of this study was: (1) to find out if the TA correlates with QoL in patients undergoing three psychotherapy modalities (psychodynamic psychotherapy, cognitive behavioral therapy, and interpersonal therapy); and secondary and third objectives were: (2) to evaluate if there is a difference in the TA and QoL correlation between these three different modalities of psychotherapy; and (3) to analyze if the correlation between TA and QoL, if positive and statistically significant, persists after controlling for confounding factors (gender, age, depressive symptoms, number of sessions, psychiatric medication use, previous suicide attempts, and previous psychiatric hospitalization).

We think it is important to evaluate the direct relationship between TA and QoL because the improvement in quality of life does not necessarily go through the improvement of symptoms, some patients may continue to have symptoms of a particular disorder but improve the way they handle them.

## Materials and Methods

The patient's perspective was the main focus of this study. A cross-sectional study, which included outpatients who were undergoing individual psychotherapeutic treatment in some of the modalities, including PP, CBT, and IPT, were selected in a specialized public outpatient clinic for mental disorders located in a tertiary hospital. Only those patients who agreed to participate in the study according to the application of the informed consent terms were included in this sample. Patients with psychotic disorders (schizophrenia, bipolar in the manic state) and drug addiction were excluded. A total of 76 patients who underwent psychotherapy (PP, IPT, and CBT) were evaluated at the psychiatric clinic of the Hospital de Clínicas de Porto Alegre (HCPA) and included in the study.

### Patient Selection

All patients who are included in the HCPA psychotherapy outpatient clinic undergo an evaluation by a senior psychiatry resident (psychotherapy residency), who determines the indication of psychotherapy under the supervision of a psychotherapy senior supervisor. Three modalities of individual psychotherapy are offered (PP, IPT, or CBT). Psychotherapeutic care is carried out by the psychiatry resident of the 2nd, 3rd, and 4th years of the residency in psychiatry under the weekly supervision of dialogued interviews of the supervisors of the Psychiatric Service. Psychiatry residents have weekly seminars on each of the offered psychotherapeutic techniques. In addition, there are weekly practical seminars when patients are interviewed by teachers that include discussions of the clinical aspects related to the cases.

### Measures

Clinical and sociodemographic variables included age, gender, religion, marital status, ethnicity, education degree, psychiatric medication use, smoking, previous psychiatric hospitalizations, previous suicide attempts, family history of psychiatric disorders, the city in which the patient lives, and with whom the patient lives.

The psychiatric profile included patient diagnosis, use of psychiatric medication, number of therapy sessions, and psychotherapy modality.

Symptoms of anxiety and depression were measured by Beck Depression Inventory (BDI) (20) and Beck Anxiety Inventory (BAI) (21); these tools are self- depression (BDI) and anxiety (BAI), respectively, developed by Aaron Beck with the aim of evaluating anxiety, suicidal ideas, and depression indices.

Therapeutic Alliance was measured with the California Psychotherapy Alliance Scales-Patient Version (CALPAS-P). CALPAS is the newest version of the California alliance scales, based on former versions of the scales (22) using factor-analyses and theoretical considerations (23, 24). Both patient and therapist versions of the scales exist, as well as later versions. In this study, we used the patient version of CALPAS (CALPAS-P). CALPAS was developed to measure four relatively independent dimensions of the alliance: (1) therapeutic alliance; (2) alliance of work; (3) understanding and involvement of the therapist; (4) agreement between the patient and the therapist in relation to goals and strategies. The 24 items of CALPAS are distributed on these four scales. Each of the CALPAS-P items receives a score ranging from 1 (absolutely no) to 7 (totally). An examination of Cronbach's alpha revealed a coefficient of 0.84 for the whole CALPAS-P scale, indicating a high internal consistency (25).

Quality of life was measured using the World Health Organization Quality of Life Instruments–Bref (WHOQOL-BREF), a quality-of-life instrument developed by the World Health Organization. This is a self-report measure and consists of 26 questions (questions one and two about general quality of life), and the answers follow a Likert scale (from 1 to 5, the higher the better the quality of life). Apart from these two questions (1 and 2), the instrument has 24 facets that comprise four domains: (1) physical; (2) psychological; (3) social relations; (4) environment (26). As a measure of the scale internal consistency, Cronbach's alpha values were acceptable (>0.7) for domains 1, 2, and 4 i.e. physical health 0.82, psychological 0.81, environment 0.80, but marginal for social relationships 0.68 (27).

All self-applied instruments were Brazilian Portuguese validated measures: WHOQOL-Bref (26), Calpas-P (28), BDI (29), and BAI (30).

Patients answered the protocol at different times of treatment. CBT patients on average responded at the beginning of treatment, IPT responded between intermediate sessions, while patients on PP varied at the time of response, with patients having responded at the beginning, middle, and end of treatment.

With respect to ethical aspects, this project is part of a larger research study. The projects have already been forwarded and approved by the Group of Research and Graduate Studies of the Hospital of Clinics of Porto Alegre (GPPG-HCPA), whose Ethics Committee is recognized by CONEP (National Research Ethics Committee) and is registered under the same number, 15-0097 (31). This is study is part of a broader project called: “Longitudinal Investigation of Psychotherapies Outcomes: LIPO study” whose research protocol is published elsewhere.

### Statistical Analysis

Even though this was a transversal study, methodologically we considered the TA as the factor under study and QoL as the outcome. Theoretically, we do not know the direction of this relationship, and it could even be bidirectional. The other variables were considered as aspects and potential confounders.

Quantitative variables were described as means and standard deviations, and categorical variables as absolute and relative frequencies. The Shapiro-Wilk normality test was conducted on all variables. Differences between demographic and psychiatric profile means in different types of therapies were calculated by Fisher's exact test and analysis of variance (ANOVA).

In order to evaluate the effect of the score on QoL domains, a generalized linear model was applied. In order to evaluate the association between these variables, the Pearson correlation coefficient was obtained. A multivariate linear regression model was used for controlling the confounding factors (number of sessions, depressive symptoms, gender, age, psychiatric medication use, previous hospitalization, and previous suicide attempt). The criterion for entering the variable in the model was a *p* < 0.20 in the bivariate analysis. The significance level was set a 5%, and the analyses were performed in the Statistical Package for the Social Science (SPSS) program version 21.0.

## Results

The composition of the sample (*n* = 76) was characterized. With respect to gender, 58 were women (76.3%), the mean age was 45 (SD = 12), 43 were married (57.3%), and most were Caucasian (85.2%). As for schooling, 35% of the sample had high school educations followed by 17% with complete University degrees and 14% with incomplete University degrees. With respect to patient distribution by therapy type, there were more patients who underwent PP [*n* = 44 (61.9%)] followed by IPT [*n* = 19 (25%)], and CBT [*n* = 13 (17%)]. PP sampling has an average of 27 sessions per patient, while IPT consisted of had five sessions, and CBT had 10 sessions per patient at the time of evaluation. There was a statistically significant difference in the mean age of patients between PP and IPT, in which CBT patients were older on average. There were also differences in the mean number of sessions between PP and CBT and also between PP and IPT. There was no difference between therapies with respect to other factors (Table 1).

**Table 1:** Sample demographic data.

	**Total (*n* = 76)**	**PP (*n* = 44)**	**IPT (*n* = 19)**	**CBT (*n* = 13)**	***P*-value (PPxIPTxCBT)**
Age mean, (SD)	45.9 (12.1)	43.1 (9.5)	47.0 (7.8)	53.1 (12.6)	0.024^a^^*^
Female sex *n* (%)	58 (76.31%)	33 (75.00%)	16 (84.21%)	11 (84.61%)	0.092^b^
Ethnicity, *n* caucasian (%)	65 (85.52.%)	37 (84.18%)	18 (94.73%)	11 (84.61%)	0.093^b^
Married, *n* (%)	43 (57.31%)	24 (54.88%)	10 (53.90%)	7 (53.84%)	0.070^c^
School degree, *n* (%):					
Elementary school	27 (35.52%)	10 (22.27%)	6 (33.65%)	3 (22.90%)	0.075^b^
High school	11 (14.47%)	23 (52.27%)	11 (57.89%)	6 (46.20%)	
College	13 (17.10%)	11 (25.00%)	2 (9.63%)	3 (23.07%)	
Mean therapy sessions (SD)	20.5 (10.4)	27.7 (15.7)	10.5 (6.6)	5.6 (5.8)	<0.001^a^^*^ 0.003^a^^**^

*M, mean; SD, standard deviation; n, number; PP, psychodynamic psychotherapy; CBT, cognitive behavior therapy; IPT, Interpersonal therapy*.

* *difference between PP and IPT sample;*

** *difference between CBT and PP sample*.

a *t-test*,

b *Fischer exact test*,

c *chi square test*.

Regarding the psychiatric profile of the patients, 20 (26.8%) had already been hospitalized in a psychiatric unit, 29 (38%) had previously attempted suicide, and 68 (89%) were using some form of psychiatric medication. As for the diagnosis of patients (reviewed in medical records), most (51.3%) had been diagnosed with depression followed by anxiety (18.4%) and bipolar mood disorder (11.8%). Attention is drawn to the severity of the depressive and anxious symptoms of the sample with a mean BDI score of 30 (severe depression) and 25 on the BAI scale (moderate anxiety). There was a statistically significant difference between the diagnostic frequencies by type of therapy, such as in IPT, in which there was a higher number of patients diagnosed with depression compared with PP, and in CBT, in which there was a higher number of patients diagnosed with anxiety when compared with PP and IPT. There was no difference between therapies with respect to the other factors (Table 2).

**Table 2:** Sample psychiatric profile.

	**Total (*n* = 76)**	**PP (*n* = 44)**	**IPT (*n* = 19)**	**CBT (*n* = 13)**	***P*-value (PPxIPTxCBT)**
Diagnosis, *n* (%)					
Depression Anxiety Bipolar disorder Personality disorder PTSD OCD Eating disorder N.D.	39 (51.31%) 14 (18.42%) 9 (11.84%) 5 (6.57%) 1 (1.31)% 1 (1.31)% 2 (2,62%) 5 (6.57%)	17 (38.63%) 6 (13.63%) 8 (18.18%) 4 (9.09%) 2 (4.54%) 1 (2.27%) - 6 (13.63%)	16 (84.21%) - 2 (10.52%) - - - - 1 (5.26%)	6 (46.15%) 5 (38.46%) - - - 2 (15.38%) -	0.039^a^^*^
Suicide attmept	29 (38.25%)	20 (45.85%)	3 (15.78%)	3 (23.07%)	0.263^a^
Previous psychiatric hospitalization	20 (26.80%)	13 (29.54%)	3 (15.78%)	3 (23.07%	0.084^a^
Pharmacological treatment	68 (89.47%)	40 (90.90%)	17 (89.47%)	12 (92.30%)	1.000^a^
BDI (mean)	30.4 (10.1)	30.7 (10.0)	27.0 (8.9)	28.5 (8.3)	0.734^b^
BAI (mean)	25.5 (12.0)	23.6 (11.3)	24.0(14.4)	25.8 (12.9)	0.053^b^

*M, mean; SD, standard deviation; n, number; BDI, Beck depression inventory; BAI, Beck anxiety inventory*.

* *Difference between IPT and PP regarding depression diagnosis, and difference between CBT and PP and CBT regarding anxiety diagnose*.

a *Fisher exact test;*

b *ANOVA*.

### Association of Therapeutic Alliance and Quality of Life

When including the total sample in the analysis, the association of the TA (CALPAS-P scale) with the QoL (WHOQOL-BREF) domains did not present a statistically significant difference (*p* < 0.05). The association of the TA with the QoL psychological domain was *b* = 1.90 (−2.66–6.46; *p* = 0.214), with the QoL physical domain it was *b* = 4.25 (−1.29–9.80; *p* = 0.527), with the QoL social domain it was *b* = 2.42 (−3.11–7.95; *p* = 0.455), and with the QoL environmental domain it was *b* = 1.45 (−2.38–7.29; *p* = 0.598). In the overall domain, which refers to the first two WHOQOL-BREF questions concerning the way in which the patient evaluates their QoL in a generic way, the correlation value was *b* = 3.22 (−2.58–5.21; *p* = 0.407) (Table 3).

**Table 3:** Association of TA (CALPAS-p score) with QoL (WHOQOL-BREF domains) in the total sample (*n* = 76) and by the psychotherapy method.

	**Total (*n* = 76) B (*p*-value)**	**PP (*n* = 44) B (*p*-value)**	**IPT (*n* = 19) B (*p*-value)**	**CBT (*n* = 13) B (*p*-value)**
Psychological	1.90 (0.214)	**7.69 (0.046)**	−11.67 (0.114)	−3.97 (0.654)
Physical	4.25 (0.527)	1.94 (0.579)	−0.829 (0.901)	1.68 (0.876)
Social	2.42 (0.455)	2.53 (0.555)	0.856 (0.905)	4.29 (0.599)
Enviromental	1.45 (0.598)	1.749 (0.589)	5.485 (0.120)	−7.76 (0.439)
Overall	3.22(0.407)	0.955 (0.851)	0.923 (0.132)	4.11 (0.694)

*CALPAS, California Psychotherapy Alliance Scale; WHOQOL, World Health Organization Quality of Life; BREF, shorter version; B, standardized beta coefficient Bold: P <0.05*.

When considering the therapies separately, there was a statistically significant in the association between the TA and the QoL psychological domain in patients undergoing PP (*b* = 7.694 (0.14–15.74; *p* = 0.046), but there were no significant differences when compared with the other QoL domains in the PP sample or in therapies in all domains (Table 4). The Pearson's correlation coefficient between the TA and the QoL psychological domain was 0.287.

**Table 4:** Confounding factors influence in PP patients, on association between the WHOQOL psychological domain and TA.

	**Gender B (*p*-value)**	**Pharmac. tratment B (*p*-value)**	**Suicidal attempt B (*p*-value)**	**BDI B (*p*-value)**	**Number of sessions B (*p*-value)**	**Previous Hosp. B (*p*-value)**	**Age B (*p*-value)**	**b (CI 95%)**
PP	**12.912 (0.023)**	15.830 (0.075)	**−12.518 (0.023)**	**−1.296 (<0.001)**	–0.013 (0.862)	4.770 (0.410)	0.187 (0.450)	2.62 (–1.58 to 6.83) *p* = 0.221

*PP, psychodynamic psychotherapy; B, standardized beta coefficient Bold: P <0.05*.

With the regression for the confounding factors, as such depressive symptoms (BDI score), number of sessions, age, gender, psychiatric medication use, previous suicide attempts, and previous psychiatric hospitalization, the association of the psychological domain with the TA in the PP patients lost significance [*b* = 2.62 (−1.58–6.83); *p* = 0.221]. The confounding factors that significantly influenced this association were gender (*b* = 12.912; *p* = 0.023), previous suicide attempts (*b* = −12.518; *p* = 0.023), and BDI (*b* = −1.296; *p* = 0.000) (Table 4).

The TA (CALPAS-P mean score) varied between therapies with 5.56 for PP, 5.98 for CBT, and 4.95 for IPT. There was no statistically significant difference among these means.

## Discussion

To the best of our knowledge, the literature review indicates that this is the first study to evaluate the association between the TA and QoL when the three modalities of psychotherapy (PP, IPT, CBT) were analyzed together. No association was found in the total sample for any WHOQOL domain. The association in the psychological and social domains was expected since those patients are supposed to be more susceptible to change after mental health treatment. It would be plausible to expect higher TA indices in those patients who present higher scores in these domains (psychological and social) of the WHOQOL-BREF. According to Lubowrsky (32), a patient's mental health is correlated with his or her ability to form an alliance, which suggests that the capacity to form an alliance is partly a quality that the patient brings to his/her treatment.

Also, this is the first study to show that there is a positive and statistically significant association in the psychological domain of the Qol measure (WHOQOL-BREF) with the TA in patients undergoing PP. This finding corroborates some theoretical assumptions because as Batista et al. (33) points out, the relationship itself is one of the most important factors in all of the psychotherapy forms (34) and is considered many times in the success of the psychotherapeutic process, yet as part of the psychoanalytic model, it is assumed that the therapeutic relationship has a much higher impact because of its main focus as a part of the therapeutic model itself. It is within the therapeutic relationship that an unconscious communication is established and developed, and on this level, changes happen during the psychotherapeutic process.

Eizirik et al. (35) corroborates this idea, saying that “Considering PP, the therapeutic relationship is even more important since the patient repeats primitive relationship patterns with the therapist, and this modality of psychotherapy allows the patients to identify and treat their maladaptive patterns”. Gomes et al. (36) also goes along this line of theoretical thinking and asserts that PP is a type of psychological treatment based on the theory and technique of psychoanalysis, but it differs from classical psychoanalysis. Both use the concept of the unconscious postulated by Freud, as well as free association and the understanding of dreams, humor, and faulty acts to understand and give meaning to unconscious conflicts. The notion that the TA is the indispensable basis of treatment based on psychoanalytic theory remains intact, and it is re-defined as a positive and stable relationship between therapist and patient, which allows us to carry out a PP (36).

In addition to the PP techniques, when the technique is based more on the therapist-patient relationship as a therapeutic process, another possible reason for the difference in the PP sample compared to the CBT/IPT sample is the characteristic of the scale used, considering that CALPAS scales are influenced by both traditional psychoanalytic concepts of the alliance and the subsequent work of Bordin (37). In 1979, Bordin (38), established a more operational definition of work alliance, consisting of goal and task agreements and patient-therapist bond development. According to this author, these characteristics are central to all psychotherapies but may develop differently in different forms of therapy (11).

Another reason for the difference between therapies may be the fact that CBT directs its efforts toward the direct reduction of symptoms; its assumption is that therapy must instrumentalize the patient to deal with his difficulties in the absence of the therapist (5). Therefore, similar to IPT, this modality of psychotherapy does not have a prerequisite to analyze and treat dysfunctional patient relationship patterns that emerge throughout the treatment in patients' relationships with their therapists. CBT and IPT goals are to teach patients some techniques to deal with their symptoms.

The association between the QoL in the psychological domain with the TA in PP patients loses significance after controlling for depressive symptoms, age, number of sessions, gender, psychiatric medication use, previous psychiatric-related hospitalizations, and previous suicide attempts. This finding can be explained partially by the contribution in which psychiatric medication use can exert symptomatic improvement (1) and consequently interfere with positive domains in the QoL and possibly in the formation of the TA; as Lubowrsky stated (32), those patients who are healthier are more able to form an alliance. Depressive symptoms are directly associated with worse/worsening QoL (17, 39), so they are a clear confounding factor in assessing the association. Therefore, patients with higher depression scores will have greater difficulty in developing the TA and engaging in treatment. Also, depression may affect the way in which they evaluate their own QoL and underestimate possible improvements.

As seen in Table 4, the patient's gender and their previous suicide attempts may also interfere with the studied association. Regarding gender, some studies have indicated that female patients tend to derive more benefit from therapy than male patients. Others have found that patients of both genders benefited more from treatment when treatment was provided by female therapists. Much of the evidence, however, suggests that the association between patient or therapist gender and treatment outcome is weak (40).

Also, there is some evidence suggesting that gender might mediate the way in which people perceive their QoL under chronic conditions, and compared to male patients, female patients have more depressive symptoms and poorer scores in the psychological QoL domain (41). In this sample, whether patients have chronic health conditions was not evaluated; however, the total patient sample (Table 2) shows that this sample population had very high depressive and anxious symptoms on average, which may affect the perception of QoL mainly in females. In addition, a study by Bonsaksen et al. (42) exploring gender differences in QoL shows that depression mediates the decrease in QOL of female psychiatric patients. Together, these factors seem to have influenced the results of the present study.

A history of previous suicide attempts was a confounding factor in the association between the TA and QoL. Some studies have shown that patients with previous suicide attempts have lower TA scores in the first treatment session, but this score increases until the third session (43). There is also evidence that those who attempt suicide tend to be less bound to treatment and consequently have a higher drop-out rate (44). The characteristics of this specific population may have influenced our study results.

Curiously, the Pearson's correlation value found in this study in the PP sample in the QoL psychological domain (*r* = 0.287) was very close to the value found in a meta-analysis evaluating TA and positive outcome in psychotherapy (*r* = 0.27) (7). However, unlike the studies included in the meta-analysis, our sample was only cross-sectional, and already in the meta-analyses, the studies were longitudinal, and the TA was studied as a possible predictor of improvement.

Our study presents some aspects that give greater robustness to the findings. Its naturalistic design provides elements of the real world (45), mainly in the context of clinical practice, such as the heterogeneous sample of patients (various diagnoses in the sample), concomitant use of medications, and inclusion of different modalities of psychotherapy make the findings of this study of particular importance. Another differential of our study was the use of WHOQOL as an outcome in psychotherapy: as we can see from recent meta-analyses, studies in psychotherapy are still very focused on symptom reduction as a response to treatment, and we understand that the concept of QoL adds a more comprehensive and humanistic view of the patient.

Another factor that strengthens the study is the use of the TA scale in the patient's version (CALPAS-P). This patient perspective is in line with the concept of the “QoL” outcome, according to WHO.

### Limitations

Although the cross-sectional design is not methodologically sophisticated enough to arrive at meaningful conclusions about the differential impact of TA on QoL across these different conditions, it doesn't discard the important findings of our study. The unique measure of the TA also has limitations because according to Horvath and Symonds (8), there is some support for the view that the alliance is not a steady state or linear phenomenon. Such regular variations in alliance levels were observed in a small-scale study. In contrast to a clinicians' integration of information across therapy, most research studies typically only use a limited sample of therapy sessions. If the alliance varies to a certain degree from session to session, sampling a single session (or even two) may provide a relatively unreliable assessment of the general status of the alliance across the entire treatment and therefore may yield results inconsistent with a clinical view of a particular therapy patient that takes into account information across all sessions (46). This study is non-randomized and has an observational and naturalistic design, and therefore does not control biases, and provides elements of the real world, mainly in the context of clinical practice.

Another important limitation of our study is the fact that we do not have a homogeneous sample of patients across several aspects, such as the timing of TA measurement, which was performed at different stages of therapy with some patients entering the study early in psychotherapy and others during more advanced stages. According to Flückiger, the relationship between alliance and outcome is higher when the alliance is measured later in therapy in comparison to the early alliance assessment (and the other alliance assessments in between these two values) (7).

In addition, the study also does not present homogeneity in the representativeness of the types of therapy, with the PP presenting more participants (40) than the CBT and IPT (32 adding the two), which makes the comparison between the therapies inaccurate. Another piece of data that undermines the comparison between therapies is the number of sessions performed at the time of evaluation; PP sampling has an average of 27 sessions per patient, while in the IPT 5 and CBT 10 sessions per patient at the time of evaluation.

Another factor that may affect our evaluation of the data collected is that the psychiatric profiles of the patients had a very high BDI average of 30 points, indicating patients with severe depressive symptoms. The provision of a caring and empathic therapeutic relationship that facilitates a strong alliance may be especially important within the context of a disorder such as depression that is often characterized by disconnection from other people, loneliness, and low self-esteem (46). Thus, the generalizability of the current findings to various outpatient populations is uncertain.

We focus on the patient's perspective on our study. Further studies, investigating the TA and QoL, should include both perspectives (therapist and patient).

### Clinical Implications

This study corroborates the philosophy that psychoanalytic theory has preached for over a century, emphasizing the importance of the therapeutic relationship in this therapeutic technique. Clearly, it is important for therapists to promote psychodynamic treatment not only in the technical aspects of their training but also to be aware of what happens at the time of the session in their relationship with the patient. This is not to say that therapists should not focus on improving techniques but to emphasize that the development and maintenance of the therapeutic relationship is a primary curative component of therapy and that the relationship provides the context in which specific techniques exert their influence (47).

### Future Perspectives

In the future, research designs are needed that can test the causal impact of the alliance along with further process variables in psychotherapy outcomes using prospective designs. More research is needed in culturally specific samples both inside and outside Western countries. More research is also needed in which the boundary conditions of the alliance measures and their interaction to interpersonal and general process indicators, such as empathy, the real relationship, and corrective experiences, are examined (7).

## Conclusion

This study shows that during analyzes of the total sample, which covers the three types of psychotherapies, there is no association between TA and QoL. However, when only patients in PP are analyzed, there is a possible association between the TA instrument and the psychological QoL domain, but this association disappears when adjusted for other confounding factors, meaning this could be driven by other factors. The association was not statistically significant and therefore can not be attributed to a relationship between the two. Future studies, especially the longitudinal ones, may point out the differences between therapies.

## Data Availability Statement

The original contributions presented in the study are included in the article/supplementary material, further inquiries can be directed to the corresponding author.

## Ethics Statement

The studies involving human participants were reviewed and approved by GPPG–HCPA no 15-0097. The patients/participants provided their written informed consent to participate in this study.

## Author Contributions

NR coordinated the project in HCPA. GB, LG, and CR implemented the project. LP was responsible for precessing data. GB and NR codeveloped the project. All authors contributed to the article and approved the submitted version.

## Funding

This study was financed in part by ‘Coordenação de aperfeiçoamento de pessoa de nível Superior – Brasil (CAPES) – finance code 001’, and another part by ‘Fundo de Incentivo à Pesquisa (FIPE/HCPA)’.

## Conflict of Interest

The authors declare that the research was conducted in the absence of any commercial or financial relationships that could be construed as a potential conflict of interest.

## Publisher's Note

All claims expressed in this article are solely those of the authors and do not necessarily represent those of their affiliated organizations, or those of the publisher, the editors and the reviewers. Any product that may be evaluated in this article, or claim that may be made by its manufacturer, is not guaranteed or endorsed by the publisher.
